# Did the COVID-19 pandemic impact urticaria information-seeking behavior in China? A retrospective longitudinal study

**DOI:** 10.3389/fpubh.2023.1098066

**Published:** 2023-01-19

**Authors:** Qinzhun Zhang, Yi Yu, Jialu He, Xinmeng Yao, Yinan He, Jinghua Wu, Chenjie Xu, Chengyin Ye

**Affiliations:** ^1^Department of Health Management, School of Public Health, Hangzhou Normal University, Hangzhou, Zhejiang, China; ^2^Department of Epidemiology and Biostatistics, School of Public Health, Hangzhou Normal University, Hangzhou, Zhejiang, China; ^3^Department of Health Policy and Management, School of Public Health, Hangzhou Normal University, Hangzhou, Zhejiang, China

**Keywords:** urticaria, Baidu index, information-seeking behavior, COVID-19 pandemic, COVID-19 vaccination

## Abstract

**Purpose:**

To investigate information-seeking behavior related to urticaria before and during the COVID-19 pandemic in China.

**Methods:**

Search query data for terms related to urticaria were retrieved using Baidu Index database from October 23, 2017 to April 23, 2022, and daily COVID-19 vaccination doses data were obtained from the website of the Chinese Center for Disease Control and Prevention. Among the 23 eligible urticaria search terms, four urticaria themes were generated as classification, symptom, etiology, and treatment of urticarial, respectively. Baidu Search Index (BSI) value for each term were extracted to analyze and compare the spatial and temporal distribution of online search behavior for urticaria before and after the COVID-19 pandemic, and to also explore the correlation between search query and daily COVID-19 vaccination doses.

**Results:**

The classification of urticaria accounted for nearly half of the urticaria queries on the internet. Regular seasonal patterns of BSI were observed in urticaria-related online search, by attaining its highest level in spring and summer and lowest level in winter. The BSIs of all urticaria themes significantly increased after the COVID-19 pandemic than that before the pandemic (all *P*<0.05). Xizang, Qinghai and Ningxia are the most active geographical areas for increased urticaria-searching activities after the COVID-19 pandemic. There was also a significant positive correlation between daily BSIs and daily COVID-19 vaccination doses in each urticaria theme. Cross-correlation analysis found that the search of symptom, etiology, and treatment attained their strongest correlation with daily COVID-19 vaccination doses at 11–27 days before the injection of vaccine, imply vaccination hesitation related to concerns of urticaria.

**Conclusions:**

This study used the internet as a proxy to provide evidence of public search interest and spatiotemporal characteristics of urticaria, and revealed that the search behavior of urticaria have increased significantly after the COVID-19 pandemic and COVID-19 vaccination. It is anticipated that the findings about such increase in search behavior, as well as the behavior of urticaria-related vaccine-hesitancy, will help guide public health education and policy regulation.

## 1. Introduction

Urticaria is a common skin condition that can manifest as wheals, angioedema, or both. It is estimated that around 20% of people will experience acute urticaria during their lifetime (1), which is usually associated with viral infections or acute allergic reactions to food, drugs, or insects (2). Among them, up to 45% of patients will progress to chronic urticaria, which is characterized by complex pathogenesis, reduced quality of life and high health care costs (3). Although two recent population-based epidemiological studies of urticaria in China reported a lifetime prevalence of urticaria of 7.30% (4) and an estimated prevalence of chronic urticaria of 2.6% (5), the actual prevalence of urticaria may be greatly underestimated given the low medical help-seeking behavior and insufficient service utilization of most urticaria patients (6, 7). Previous studies have shown that 76.9% of adults use the internet for health purposes (8), and many patients tend to use internet searches to seek for initial diagnosis and health advice/solutions. In addition, online search behavior has been proved to be correlated with disease epidemic trends or actual related events (9–11). Thus, we speculated that patients with urticaria tend to use the internet as a common source of health information to initiate their information-seeking behaviors for self-care rather than directly seeking professional help, especially those with milder symptoms or less frequent episodes (12, 13). In this context, internet search analysis could provide a spatial-temporal mapping of the interest and medical needs of the internet-using population or potential patients on the specific theme of urticaria.

Meanwhile, the Coronavirus disease 2019 (COVID-19) pandemic has severely disrupted healthcare systems and posed serious challenges to patient care, leading to multiple direct and indirect health impacts and social burdens (14, 15). On the one hand, innate and lifestyle-related factors, such as age, physical activity, and health status, were influential factors of the incidence and severity for COVID-19 (16). It was found that the severity of infection was negatively correlated with the acceptance (adherence and respect) of preventive measures, suggesting that lifestyle-related factors are more important than innate factors. On the other hand, COVID-19 also largely disrupts patient referrals for emergency medical care and essential health services provision, which should be considered as a major health threat that needs to be addressed during the COVID-19 pandemic (15, 17). It has been reported that the COVID-19 pandemic may be associated with urticaria in the general population in different ways. Firstly, urticaria has been reported as a common symptom of COVID-19 patients in the course of the disease after infection (18–20). Meanwhile, many drugs used to treat COVID-19 can cause urticaria, such as remdesivir, chloroquine, and lopinavir (21). Secondly, along with the progress of the vaccination program in response to the COVID-19 pandemic, part of vaccinators self-reported at least one local or systemic side effect, such as injection site pain and heat, arm pain or delayed systemic urticarial reactions (22, 23). Moreover, compared with general population, atopic patients developed significantly more fever, nausea and vomiting, as well as skin rash (urticaria) (24). These adverse effects resulted in public concerns and fears about side vaccine reactions before vaccination (vaccine hesitation) (25). Thirdly, psychosocial stress caused by the COVID-19 pandemic's prolonged social constraints may also trigger or aggravate urticaria (26). At the same time, internet use has rapidly increased due to social distancing norms and nationwide lockdowns, more importantly, the epidemic has also triggered a growth of the public's health awareness and the demand for authoritative medical knowledge (27). Therefore, it can be anticipated that searches for information related to urticaria will increase greatly during the COVID-19 pandemic. The most popular search engine tool that uses Internet-based search data to analyze health problems and behaviors is Google Trends and the most successful example is the use of Google Trends by Ginsberg et al. to predict the occurrence of influenza (28). In mainland China, Baidu is the most commonly used search engine, with nearly 86% of the country's internet users accessing it (21). Baidu search index (BSI) is a big data analysis platform with various visual interfaces that takes the exact matching keywords searched by a large number of Baidu users over a period of time as the statistical object. The algorithm generates data by first referring to the number of searchers for a certain keyword in Baidu web search, and then analyzing and calculating the weighted sum of the number of searches, providing users with final BSI values (29, 30). Thus, the Baidu search index can be used to investigate the online health information search behavior and to reflect the real-world health needs of Chinese internet users and has been successfully applied in studies of COVID-19 and traditional infectious diseases outbreak surveillance and prediction, cancer investigations, and urological diseases, eye diseases, and allergic diseases online attention (11, 30–34). Moreover, according to “2020 National Health Search Data” released by Baidu Health, “Urticaria” became one of the top 10 most popular search terms for diseases after “COVID-19” (27), indicating that urticaria was the non-communicable disease obtaining the highest public attention during the COVID-19 pandemic.

This study aimed to compare changes in information-seeking behavior related to urticaria before and after the COVID-19 pandemic in terms of search volume, as well as explore the correlation between urticaria-related search behavior and COVID-19 vaccination progress in China.

## 2. Materials and methods

### 2.1. Keyword selection and web search data retrieval

**Figure 1:**
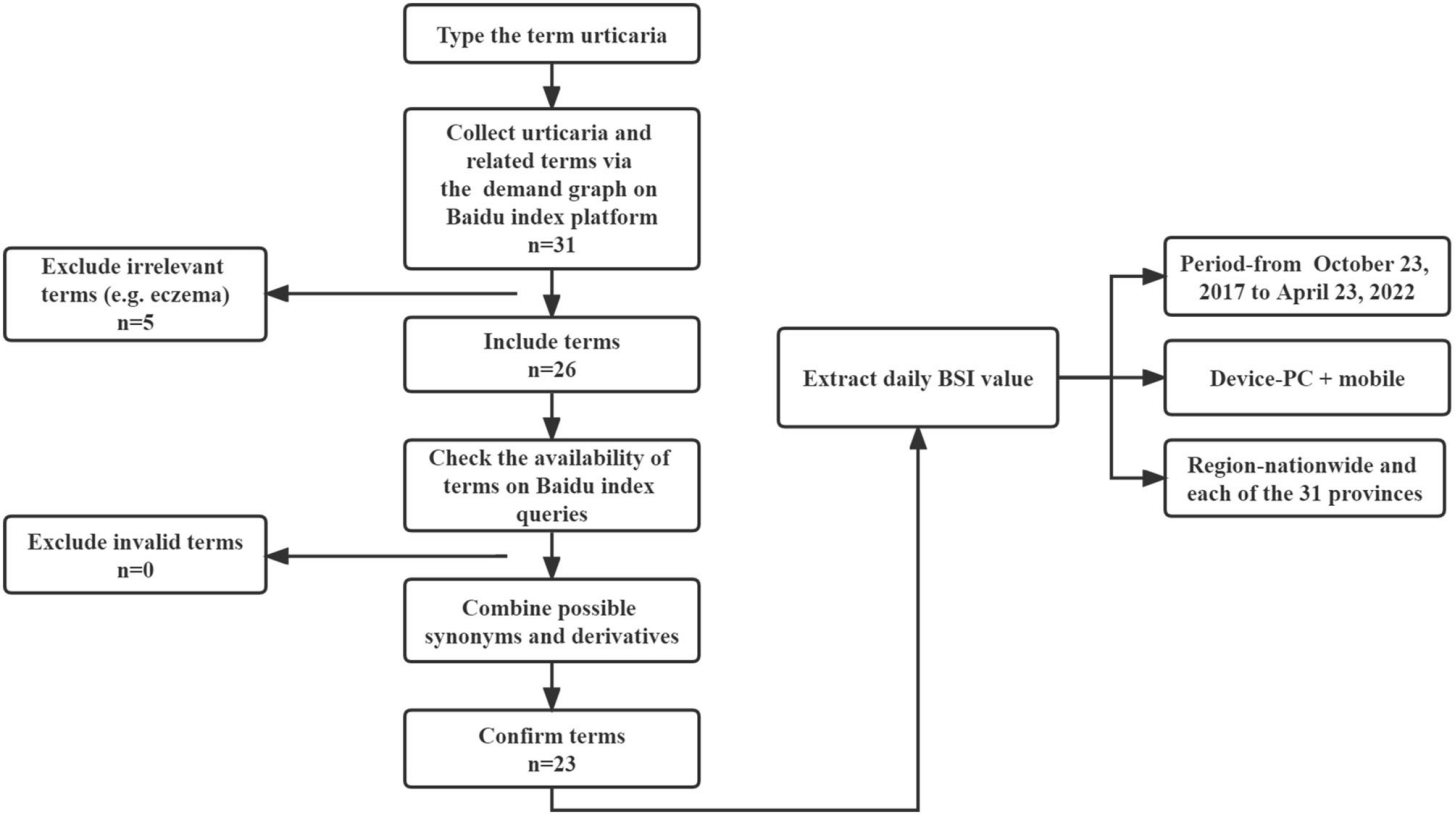
Flowchart for searching terms identification.

In this study, the keywords were generated by referring to the demand graph of “urticaria” on the Baidu index platform for the one-year period from April 26, 2021 to April 24, 2022. The demand graph automatically gathered the top search terms related urticaria from Baidu web search over the past 1 year. Since BSI was generated based on exact matching of keywords, about 31 unique keywords were initially extracted from the demand graph. Then, all possible synonymous and derivative keywords were screened and combined in order to reduce nuances and biases related to linguistic and colloquial usage habits (11, 33). As a result, a final collection of 23 urticaria-related keywords were formed for the following analysis (Figure 1). According to the international guideline for urticaria and experience of medical professionals (1), the 23 urticaria-related keywords could be further classified into 4 urticaria themes, which were classification of urticaria, symptom of urticaria, etiology of urticaria and treatment of urticaria. All included urticaria keywords are listed in Supplementary Table 1. The BSI values were extracted for each keyword at both national and province-levels, which were the standardized daily absolute value of the search volume of the keyword in the Baidu web search (33).

On January 23, 2020, China implemented the first lockdown in Hubei Province, which was recognized as a landmark event in the outbreak of the COVID-19 pandemic in China (35). Therefore, in this study, to match the time span before and after the “COVID-19” pandemic, the “pre-COVID-19 pandemic phase” (*n* = 822 days) was defined as a time period between October 23, 2017 and January 22, 2020, while the “post-COVID-19 pandemic phase” (*n* = 822 days) was defined as the time period between January 23, 2020 and April 23, 2022. Moreover, given that the first batch of China's COVID-19 vaccination began on January 1, 2021, the “pre-COVID-19 vaccination phase” (*N* = 344 days) was defined as the period from January 23, 2020 to December 31, 2020, while “post-COVID-19 vaccination phase” (*N* = 478 days) was labeled as the period from January 1, 2021 to April 23, 2022. In summary, the time period of our study (October 23, 2017 to April 23, 2022) was divided into three sections: (1) October 23, 2017 to January 22, 2020 (pre-COVID-19 phase) (2) January 23, 2020 to December 31, 2020 (pre-COVID-19 vaccination phase) and (3) January 1, 2021 to April 23, 2022 (post-COVID-19 vaccination phase).

For data extraction, we obtained the daily BSI values for each keyword from October 23, 2017, to April 23, 2022 from the Baidu Index platform. The daily BSI for each urticaria theme was calculated as the sum of all keyword searches under the same theme per day. The total daily BSI was the sum of the daily search volume for all keywords. After that, we aggregated the daily BSI into the monthly BSI for each keyword and theme.

### 2.2. CCDC data and netizen data

To obtain vaccination data, we collected cumulative daily vaccination data from the COVID-19 Vaccination Daily Reporting System, launched by the Chinese Center For Disease Control And Prevention (CCDC) on March 23, 2021 (36). Based on this, daily net COVID-19 vaccination data was calculated since March 24, 2021. When exploring the search behavior related to urticaria before and after the pandemic in China as well as 31 provinces, the impact of the pandemic prevention and control measures on increased Internet access should be considered. Therefore, we extracted the number of Internet users for the year of 2019 and 2020, respectively from the China Statistical Yearbook-2020 (37) and China Statistical Yearbook-2021 (38) to calculate the adjusted daily BSI averages. The adjusted daily BSI average (before the pandemic) = daily BSI/number of Internet users in 2019 (millions), while the adjusted daily BSI average (after the pandemic) = daily BSI/number of Internet users in 2020 (millions). As a result, adjusted daily BSI average represented the daily BSI searches per million Internet users before and after the pandemic, which was further used to compare the difference in search volume before and after the pandemic. Please note that China Statistical Yearbook-2022 has not been published yet, so the number of Internet users in the year of 2021 was unavailable at the time.

### 2.3. Statistical analysis

We compiled daily BSI for each urticaria topic from October 23, 2017 to April 23, 2022, and used R software's ggplot2 package to visualize and compare time trends between total daily searches and 4 individual themes of classification, symptom, etiology, and treatment, using line plots (39). The time-series seasonal decomposition analysis based on the seasonal trend decomposition using Loess in R was used to understand the seasonal patterns and long-term trends of the 53-month total urticaria search volume and the four urticaria themes from November 2017 to March 2022 (40). The time series of BSI were divided into seasonal, trend and residual components.

Mann-Whitney *U*-test was used to compare the daily BSIs for different themes of urticaria between different time periods. Spearman's correlation coefficient was used to measure relationships between the daily BSI of each urticaria theme and daily net doses of COVID-19 vaccination during the time period between March 24, 2021 and April 23, 2022. Cross-correlations were further adopted to determine the time-lag between the daily search activity and daily doses. Time series cross-correlation functions (CCFs) were carried out to compare the explanatory (i.e., daily COVID-19 vaccination doses) and dependent (i.e., daily BSI of each theme) time series variables. Lags of the time series were determined by comparing asynchronous cross-correlations and synchronous cross-correlations. The correlation coefficient, *r*, was interpreted as follows: *r* ≤ 0.2~0.1 (very weak), *r* ≥ 0.3~0.5 (fair), *r* ≥ 0.6~0.7 (moderate), *r* ≥ 0.8~0.9 (very strong), and *r* = 1 (perfect) (41). The significance of CCF was determined under *P* < 0.05. The Mann-Whitney *U*-test were conducted using IBM SPSS, version 26.0 (IBM Corporation) (42), and other statistical analyses and plots were conducted in R software (version 4.1.1).

## 3. Results

### 3.1. Description of the overall spatiotemporal change trend of web-based search in urticaria themes

**Figure 2:**
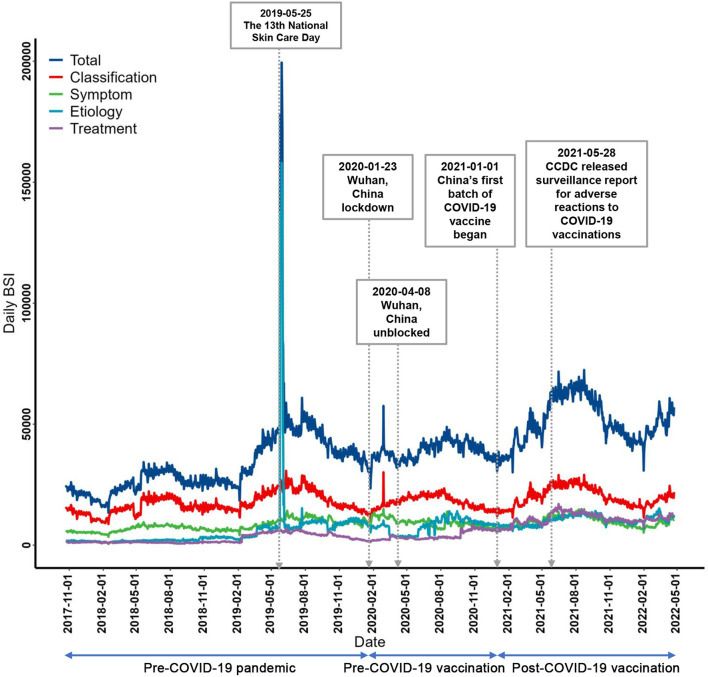
Daily BSIs comparison in urticaria search themes (including the total search) from October 23, 2017 to April 23, 2022.

In the time period from October 23, 2017, to April 23, 2022, the total BSI value of urticaria-related search terms was 64,961,359. The BSI for each urticaria theme varied widely. The classification theme mainly involves the terminology of urticaria subtypes and was the dominant component, accounting for nearly half of the total urticaria searches (*n* = 29,700,202, 45.72%), and the symptom-related searches (*n* = 14,328,623, 22.06%) occupied the second place, while the etiology (*n* = 12,352,287, 19.01%) and treatment themes (*n* = 8,580,247, 13.21%) accounted for < 20% of the total search, respectively (Table 1). The daily BSIs for the total search and the 4 themes were presented in Figure 2. Specifically, the classification and symptom theme followed each other and shared a similar increase pattern, as their daily BSI revealed a gentle upward and seasonal trend throughout the study period. On the other hand, the theme of treatment showed a smooth pattern at first but had a relatively large increase after November 2020. Notably, during the Wuhan blockade, there was a transient small peak in daily BSI for classification, symptom, and etiology themes of urticaria. In addition, there were two large peaks in daily BSI for almost each urticaria theme during the study period (Figure 2). The first notable spike was found for the theme of etiology during the last week of May, 2019, and this mainly resulted from a surge in searches for the term “Is urticaria contagious” (Supplementary Figure 1) this was the week that a nationwide skin care awareness campaign was launched following public health awareness day “May 25 National Skincare Day”. The second surge occurred after the CCDC released the COVID-19 Vaccination Adverse Reaction Surveillance Report on May 28, 2021.

**Table 1:** The BSIs of the total search and four themes of urticaria from October 23, 2017 to April 23, 2022.

**Theme**	**BSI (*n* = 64,961,359), *n* (%)**
Classification	29,700,202 (45.72)
Symptom	14,328,623 (22.06)
Etiology	12,352,287 (19.01)
Treatment	8,580,247 (13.21)

**Figure 3:**
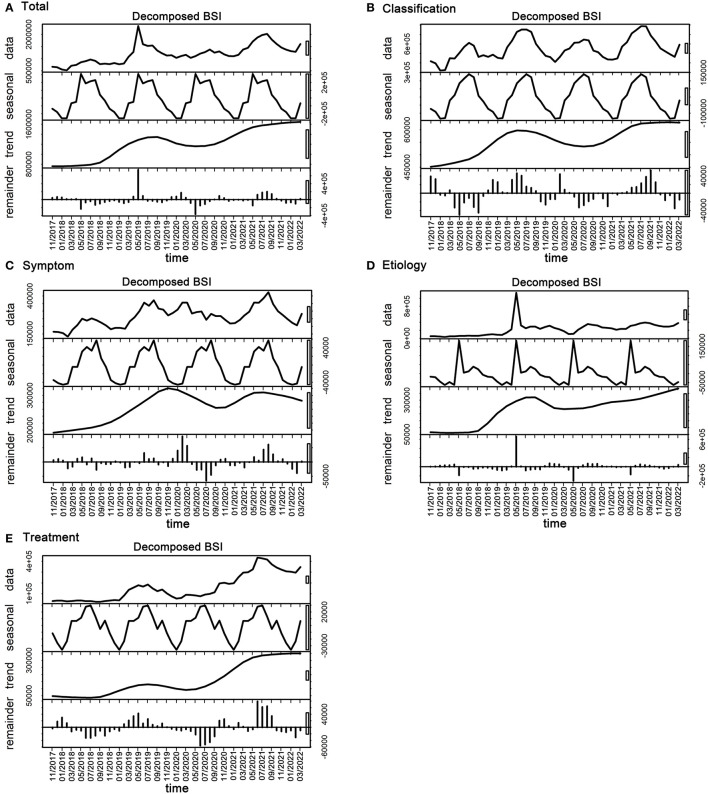
Seasonal and trend decomposition for the monthly BSIs of **(A)** the total search and the four themes: **(B)** Classification, **(C)** Symptom, **(D)** Etiology, and **(E)** Treatment, from November 2017 to March 2022.

Furthermore, in order to identify seasonal patterns and more importantly explore the remaining trends of searching behaviors related to urticaria terms after removing seasonal effects over the study period, we applied the time series decomposition algorithm on the monthly BSI patterns of the total search and the four themes of urticaria from November 2017 to March 2022 (Figure 3). Both annual and seasonal patterns were observed in all 4 urticaria themes. Regarding the seasonal fluctuation, except for the symptom theme that attained the highest BSI in August (summer), all of the rest 3 themes reached their highest BSIs in May (spring) or July (summer). On the other hand, January or February (winter) has the lowest BSIs for all themes (Supplementary Figure 2). As for the trend component, there was generally an increasing trend in BSI of the overall searching behaviors related to urticaria. Specifically, BSIs of the classification, etiology and treatment components reached a small peak in 2019, but declined in the first half of 2020 and began to rise again in the last quarter of 2020. It was worth noting, after the COVID-19 pandemic, the BSI of the symptom theme showed a second peak between May and July of 2021, while the other 3 urticarial-related themes all showed a continued upward trend.

**Figure 4:**
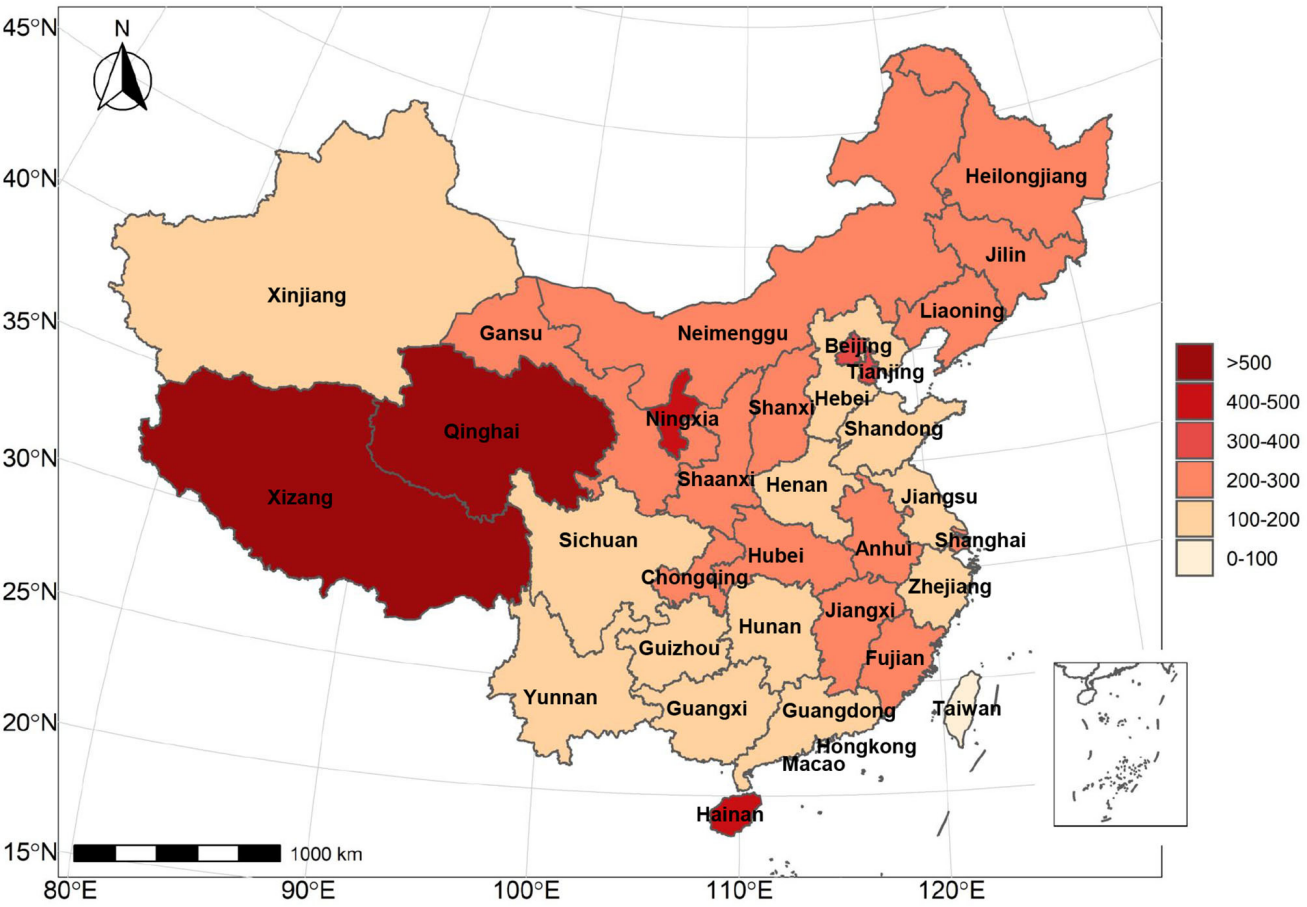
The spatial distribution of average BSI changes before and after COVID-19 pandemic. Pre-COVID-19 pandemic period: from October 23, 2017 to January 22, 2020. Post-COVID-19 pandemic period: from January 23, 2020 to April 23, 2022.

To explore the difference of BSIs of urticaria search terms in different geographical regions, we further collected the total BSI of all search terms in 31 provinces of China individually during pre-COVID-19 pandemic phase (From October 23, 2017 to January 22, 2020) and post-COVID-19 pandemic phase (From January 23, 2020 to April 23, 2022). Before COVID-19 pandemic, the top 5 provinces with relatively high overall searches were Guangdong, Jiangsu, Shandong, Zhejiang and Henan, while after the COVID-19 pandemic, Sichuan province replaced Zhejiang, and became the new top 5 BSI provinces for more urticaria searches (Supplementary Table 2). After considering the variation of population density and the difference in the number of internet users among different provinces, we calculated the adjusted BSI average of each province using the following equation: the adjusted BSI average = the overall BSI/internet users. The changes of the adjusted BSI average before and after COVID-19 pandemic were shown in Figure 4, and the top 6 provinces with the largest increase of adjusted BSI average were Xizang, Qinghai, Ningxia, Hainan, Tianjin, and Beijing.

### 3.2. Comparison of BSI related to urticaria in different stages of COVID-19 pandemic

There is growing evidence that not only can COVID-19 infection trigger symptoms of urticaria during the pandemic, but studies have reported that symptoms of urticaria may also occur after COVID-19 vaccination. To investigate changes in public attention and interest in information about urticaria during different phases of the COVID-19 pandemic, the adjusted daily BSI averages of the 4 themes of urticaria were compared between the 2 years before and after the pandemic using the Mann-Whitney *U*-test. The results showed that, after the outbreak of COVID-19 pandemic, the adjusted daily BSI averages were significantly higher for all urticaria themes (all *P* < 0.05) than that of the pre-COVID-19 pandemic period (Table 2). Moreover, we compared the adjusted daily search volumes of urticaria themes in the 1-year period before and after the start of COVID-19 vaccination, and they were also increased significantly after the start of the national-wide COVID-19 vaccination program for all the 4 themes (all *P* < 0.05) than those in the pre-COVID-19 vaccination phase after the pandemic.

**Table 2:** The comparison of adjusted daily BSI averages of urticaria before and after COVID-19 pandemic and COVID-19 vaccination.

**Theme**	**2018/1/23–2020/1/22 (*n* = 730)**	**2020/1/23–2022/1/23 (*n* = 731)**	**Z**	***P*-value**	**2020/1/1–2020/12/31 (*n* = 366)**	**2021/1/1–2021/12/31 (*n* = 365)**	**Z**	***P*-value**
	**Pre-COVID-19 pandemic**	**Post-COVID-19 pandemic**			**Pre-COVID-19 vaccination**	**Post-COVID-19 vaccination**		
	**Median (IQR)**	**Median (IQR)**			**Median (IQR)**	**Median (IQR)**		
Classification	12.64 (4.17)	13.86 (4.01)	−5.382	< 0.05	13.01 (3.38)	15.17 (4.94)	−9.955	< 0.05
Symptom	5.97 (2.36)	7.01 (2.32)	−11.778	< 0.05	6.91 (1.65)	7.36 (2.49)	−5.218	< 0.05
Etiology	2.48 (4.64)	6.83 (2.34)	−20.639	< 0.05	6.31 (2.39)	7.51 (2.79)	−9.069	< 0.05
Treatment	1.07 (2.48)	5.17 (5.64)	−23.571	< 0.05	2.25 (2.35)	7.94 (2.68)	−22.755	< 0.05

### 3.3. Correlation between BSI of urticaria and the daily doses of COVID-19 vaccination

**Figure 5:**
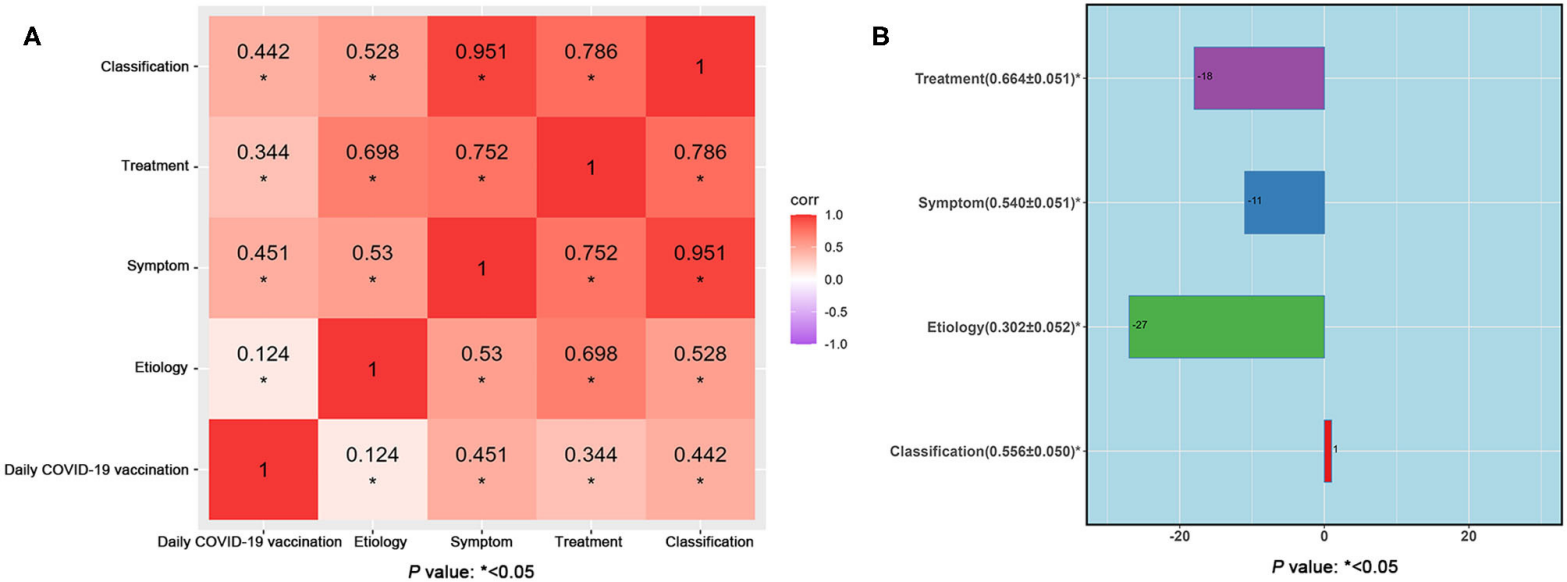
**(A)** Correlation coefficients between BSI in each urticaria theme and daily COVID-19 vaccination between March 24, 2021 and April 23, 2022. **(B)** The highest cross-correlation function of BSI with daily COVID-19 vaccination at respective time lag between March 24, 2021 and April 23, 2022.

By using the correlation analysis, it was found that the online searches for urticaria were significantly correlated with the daily doses of COVID-19 vaccination in China. Figure 5A showed a Spearman correlation between BSI in each urticaria theme and the daily doses of COVID-19 vaccine, where the strongest correlation was observed between urticaria symptoms and the daily doses (*r* = 0.451), followed by classification (*r* = 0.442). Treatment showed a fairly positive correlation (*r* = 0.344), whereas etiology had the weakest correlation (*r* = 0.124). In details, the top-5 strongest positively-correlated terms with daily doses of COVID-19 vaccine were “Pictures of early symptoms of urticaria” (*r* = 0.587, *p* < 0.05), “Chronic urticaria” (*r* = 0.555, *p* < 0.05), “Acute urticaria” (*r* = 0.543, *p* < 0.05), “Five foods you can't eat when having urticaria” (*r* = 0.499, *p* < 0.05), and “Urticaria” (*r* = 0.402, *p* < 0.05) (Table 3).

**Table 3:** The top-5 strongest correlation between daily BSIs of urticaria-related search terms and the daily doses of COVID-19 vaccination between March 24, 2021 and April 23, 2022.

**Urticaria-related search terms**	**Theme**	**Spearman's rho**	***p*-value**
Pictures of early symptoms of urticaria	Symptom	0.587	< 0.05
Chronic urticaria	Classification	0.555	< 0.05
Acute urticaria	Classification	0.543	< 0.05
Five foods you can't eat when having urticaria	Treatment	0.499	< 0.05
Urticaria	Classification	0.402	< 0.05

Finally, the cross-correlation analysis was adopted to reveal the time lag between the daily BSI of each urticaria theme and the daily doses of COVID-19 vaccine, and the largest value of correlation ± SE (*r* ± SE) was considered for interpretation of a time lag (days), where a negative lag indicated that the term was searched prior to the COVID-19 vaccination while a positive lag indicates a searching behavior after the vaccination. As shown in Figure 5B, the urticaria treatment theme searches attained its strongest correlation with vaccination doses (*r* ± SE = 0.664 ± 0.051) at 18 days before than the time of actual injection. Urticaria symptom theme and etiology theme searches were fairly correlated with daily COVID-19 vaccination doses, reaching their highest correlation at 11 days (*r* ± SE = 0.540 ± 0.051) and 27 days (*r* ± SE = 0.302 ± 0.052) before the injection of vaccine, respectively. These above findings might be associated with concerns/worries that vaccination could trigger urticaria, termed as vaccination hesitation. However, the urticaria classification theme reached a positive lag of 1 day (*r* ± SE = 0.556 ± 0.050), indicating that an explosive search behavior on the first day after the injection of vaccine.

## 4. Discussion

### 4.1. Principal findings

To our knowledge, this is the first infodemiological study investigating the online search behavior of the Chinese public for urticaria. Our results revealed that there was a significant increase in trends of urticaria-related search after the COVID-19 pandemic, along with stable seasonal fluctuations. Furthermore, online search activity for urticaria continuously increased after the national-wide COVID-19 vaccination in China, which might be partly associated with COVID-19 vaccine hesitation involving concerns of potential reactions or side effects of vaccination.

### 4.2. Main themes of interest and medical needs

Our results indicated that Baidu users' search volume and search trends for different themes of urticaria varied widely. The classification theme accounted for nearly half of the urticaria queries, suggesting that the primary goal of people's searching behavior on the internet is to find a clearer and specialized definition of various urticaria subtypes, as most people had only layman's medical knowledge and cannot fully understand medical terminology of urticaria in the first place. Another relevant study demonstrated that such online search of specialized medical phrases often occurred after a patient's diagnosis (43). In addition, when entering search terms, Baidu, like most search engines, automatically lists the most frequently searched keywords and the high-ranking website, potentially leading users to search for a set of relevant queries simultaneously. For the symptom-related terms, the general public searched the internet for pictures and symptoms of urticaria to match their own symptoms and distinguish them from common skin allergies or other skin diseases, revealing that people were tend to use the internet for self-diagnosis. The search volume of treatment-related theme, although were relatively low among the four themes during the time span of this study, also showed a dramatic fluctuation and growth after the pandemic. This indicated that a growing number of people are using the Internet to access treatment-related information for self-care and health management, which may be associated with the reduction or adjustment of outpatient services of clinics and hospitals during the COVID-19 pandemic (44).

We noticed that during the week of 2019 China's National Skin Care Day (from May 25, 2019 to May 31, 2019), the search volume of the etiology theme increased sharply, which was exclusively caused by a dramatic search increase in the term of “Is urticaria contagious”. After checking if there were any hot events at that time, we found that a popular science article was published on the “Youlai Doctor” website that week, with the exact same title “Is urticaria contagious?” (45). “Youlai Doctor” is one of the most popular knowledge-sharing platforms in the medical field in China, which mainly focuses on serving well-known experts in top hospitals and producing authoritative medical and health-related science knowledge (46). During the week of 2019 China's National Skin Care Day, the platform released an article called “Is Urticaria Contagious?”, written by an authoritative medical expert from the First Hospital of Peking University. The article, which was published together with an easy-to-understand video product, has been viewed for 114,605 times since then, far more than most articles of urticaria on the website (45). Given the authority and popularity of both the website and the article, we speculated that this event directly triggered a large amount of active online searches for the term “Is Urticaria Contagious” on the Internet, resulting in the sharp increase. This also reflects that, the public has high demand and interests of disease knowledge and skills related to self-care and self-protection, which can be easily activated if medical professionals educate them in an appropriate way, among which online education should be considered as a good choice.

However, in a review of online resources for urticaria, it has mentioned that only a few urticaria online resources were found to be readable and of high quality, while more than half were of poor or extremely poor quality. Several websites had high-quality content, but they required college-level reading/comprehension skills, which might not be suitable for all patients (47). Therefore, authorities and medical associations should take action to improve the quality of online urticaria resources and keep reviewing and updating these sites to ensure that patients have access to reliable, understandable and high-quality health information resource.

### 4.3. Temporal and spatial attention differences related to COVID-19

Our study showed that regarding the seasonal variation of BSI in urticaria, searching peaks usually occurred in spring and summer, and the curve returned to low levels in winter, which was in line with the results of previous epidemiological studies (48–50). This seasonality of public attention could be attributed to environmental or seasonal triggers of urticaria symptoms, such as allergen exposure and climatic conditions. For instance, pollen and dust mites are reported to be common allergens in spring that can provoke urticaria through airborne sensitization. The trend of summer spikes has also been observed in previous studies, suggesting the influence of a variety of factors, including temperature, humidity, and insect bites (49, 51). In addition, behavioral lifestyle may be the primary driver behind the seasonal pattern of urticaria. Significant differences in summer and winter dressing make skin exposure an important risk factor of urticaria, driving patients to seek healthcare rather than the seasonality of the disease process (52). In conclusion, spring and summer are the seasons with the highest incidence of urticaria and should be monitored carefully for the incidence of urticaria and medical needs of patients.

In terms of the trend component, which was calculated by removing the regular seasonal patterns from the original BSI, the total search behavior related to urticaria generally showed an upward trend. From the GBD (Global Burden of Disease) database, it was reported that the number of urticaria cases and the number of patients increased year by year from 2017 to 2019, which was consistent with the search trend of urticaria in our study (53). Specifically, online searches for urticaria declined in the first half of 2020, but began to rise again since the last quarter of 2020, rapidly surpassing search volumes of most urticaria themes in 2018 and 2019. In summary, the search volume of urticaria was significantly higher after the pandemic than that before the COVID-19 pandemic. As previous studies have shown, people suffered from urticaria more frequently after the outbreak of COVID-19 (54, 55). Our finding of more active online information seeking behavior was in line with the hypothesis of increased incidence of urticaria following the outbreak of the COVID-19 pandemic. Cross-reaction between viral IgM/IgG and mast cell IgE to cause mast cell degranulation could be used to explain the comorbidity of urticaria-like lesions and COVID-19 infection (56). Furthermore, ongoing psychosocial stress related to the current pandemic may further contribute to the exacerbation or onset of urticaria, which may involve stress-induced immunity decline (26).

From the geographic data, we found that the provinces and municipalities with the largest increase in averaged BSI before and after the COVID-19 pandemic were Xizang, followed by Qinghai, Ningxia, Hainan, Tianjin, and Beijing. According to the case report of COVID-19 on the official website of the National Health Commission (57) and the bulletin of the China's 7th national population census (2020) (58), we estimated the annual incidence rate of COVID-19 in 2020 and 2021 in the top-6 provinces and municipalities with the largest increase in urticaria search volume. The annual incidence rate of Xizang, Qinghai, Ningxia, Hainan, Tianjin and Beijing in 2020 was 0.27, 3.04, 10.41, 16.96, 22.28, and 45.08 per million population, respectively, and the annual incidence rate in 2021 was 0, 2.03, 6.53, 1.88, 20.19 and 10.23 per million population, respectively (Supplementary Table 3). As the incidence rate varies greatly among these regions, we cannot conclude that the increase in search volume of urticaria is significantly associated with the severity of the COVID-19 pandemic. On the other hand, these geographical differences may be explained by geographical location, climatic factors, as well as cultural background. Interestingly, our findings on the geographic differences of online search for urticaria are generally aligned with a prior study of China's public attention about the Ebola epidemic (59). Compared to eastern coastal cities, Xizang, Qinghai and Ningxia are among the more socioeconomically deprived minority regions. Social determinants of health, including less developed economies, poorer healthcare accessibility, the lack of trained medical personnel, and inconvenient transportation, have led people in these regions to seek information more frequently on the internet rather than consulting local outpatient physicians (59). On the other hand, Hainan has a lower latitude and hot and humid climate, and thus had increased prevalence of urticaria (51). Beijing, China's political and cultural hub has also been highly concerned about urticaria during the COVID-19 pandemic. Unlike users in poorer areas in the southwest, most people in Beijing are more likely to search the internet for research and educational purposes as Beijing is home to the country's largest number of top universities. In summary, the regional differences in the search volume index can provide reference information for local government decision-making and targeted regional health education programs.

### 4.4. Online searching behaviors related to COVID-19 vaccination

We also noted that there was a moderate correlation between BSI of urticaria and daily COVID-19 vaccination dosage. The greatest correlation was observed between daily dosage and urticaria symptoms, and the most positively correlated search term was “Picture of early signs of urticaria” (*r* = 0.587, *p* < 0.05). Interestingly, the second peak of symptom-related searches occurred in May 2021, which is exactly when the CCDC released the COVID-19 Vaccination Adverse Reaction Surveillance Report. In the report, some allergic skin symptoms similar to or related to urticaria (e.g., allergic rash and angioedema) were mentioned as possible side effects of vaccination. Public concerns about side effects or adverse skin reactions to COVID-19 vaccination (25) and this perceived risk may lead to vaccine hesitation, especially in patients with a history of urticaria (60, 61). This was proved by our cross-correlation analysis that the search of symptom, etiology and treatment attained their highest correlation with COVID-19 vaccination doses at 11–27 days before the injection of vaccine. During the COVID-19 pandemic, the number of patients visiting dermatological clinics were merely the tip of the iceberg, as people were reluctant to visit clinics unless they have a severe disease/condition (62). Therefore, the Baidu index can be used as a powerful tool to characterize and monitor users' online information search behavior on urticaria themes during the pandemic, and to some extent reveal potential public demands related to the diagnosis and treatment of the disease.

### 4.5. Limitations

Our study has several limitations. Firstly, the BSI only derived from search data of Baidu platform, which may ignore online search trends that exist in other Chinese search engines (such as Sogou, 360, etc.,) and social media platforms, as well as data from other Web-based sources and tools. Secondly, since the demand graph on the Baidu index platform only provides a maximum of 1 year of top search queries, previously important search terms may be ignored in our study. Thirdly, the demographic data, such as age, gender, ethnicity, education status and income, were currently not available on the Baidu index, making detailed subgroup analysis of search data impossible.

## 5. Conclusion

The search volume of urticaria-related terms increased significantly in recent years, especially after the COVID-19 pandemic and the COVID-19 vaccination. Our findings help explain the COVID-19 vaccine hesitancy related to allergic side effects involving urticaria, revealing the importance and necessity of targeted and appropriate personalized vaccination education/promotion to people having such concerns. It is hoped that by using the internet as a proxy, this study findings will enable health care professionals to better understand the changing public interests of urticaria after the COVID-19 pandemic, as well as to better respond to unmet medical needs related to urticaria care in the real world.

## Data availability statement

The original contributions presented in the study are included in the article/Supplementary material, further inquiries can be directed to the corresponding author.

## Author contributions

QZ collected the data, carried out the initial analysis, interpretation of data, and drafted the initial manuscript. YY, JH, and XY provided comments, proofreading, reviewed, and revised the manuscript. YH and JW provided comments, reviewed, and revised the manuscript. CX provided comments and reviewed the manuscript. CY contributed to the study conceptualization and design, the detail discussion, manuscript reviewing, and modification. All authors have read, approved this submission for publication, and agreed to be accountable for all aspects of the work.
