# Supplementary material for: Did the COVID-19 pandemic impact urticaria information-seeking behavior in China? A retrospective longitudinal study
Source: Front Public Health. 2023 Jan 19;11:1098066. doi: 10.3389/fpubh.2023.1098066 (PMC9894563; doi:10.3389/fpubh.2023.1098066)
Supplement: Supplementary file 1 [file Data_Sheet_1.pdf]

Supplementary Material

Supplementary Figures

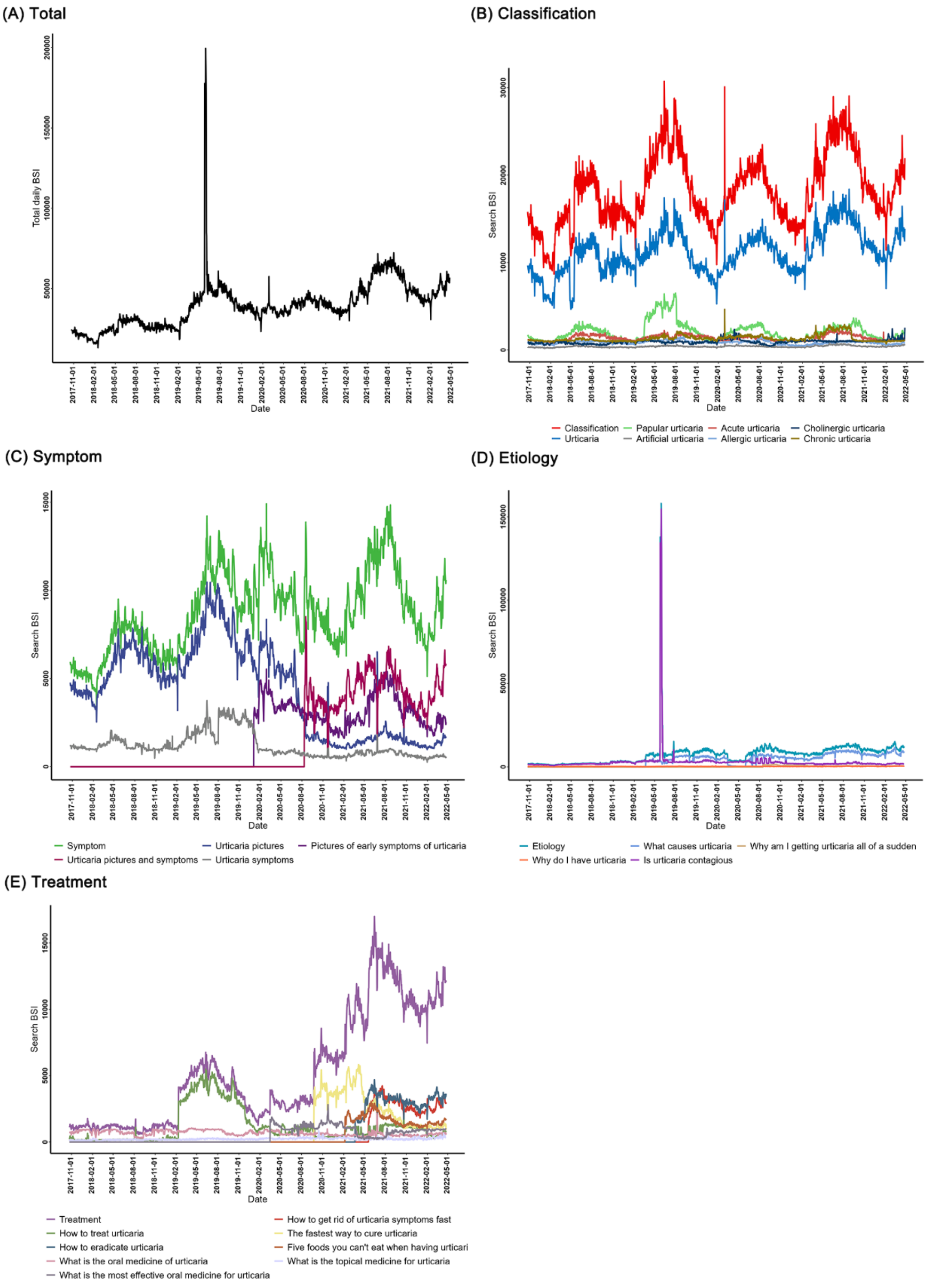

Supplementary Figure 1. The summed search trend and detailed search trend of the individual urticaria search keywords in (A) the total search and the four themes: (B) Classification, (C) Symptom, (D) Etiology and (E) Treatment, from November 2017 to March 2022.

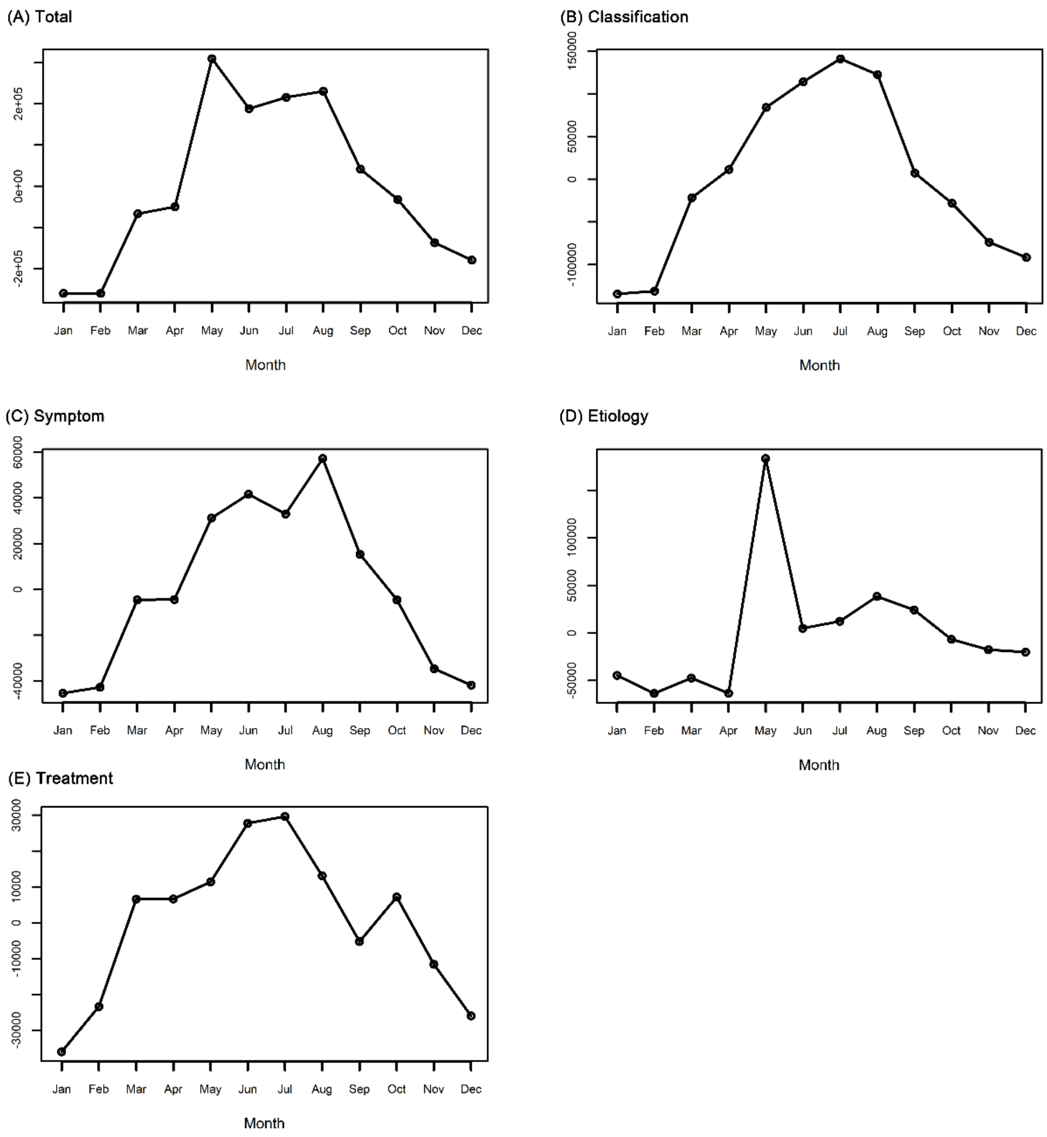

Supplementary Figure 2. Seasonal variation components of the respective monthly BSI for (A) the total search and the four themes: (B) Classification, (C) Symptom, (D) Etiology and (E) Treatment, from November 2017 to March 2022. The Y-axis represents the decomposed seasonal component data.

Supplementary Tables

Supplementary Table 1. List of 23 searching keywords used in compositing search index.

Theme of terms	Available term in Search engine	English equivalent terms
Classification	荨麻疹	Urticaria
	丘疹性荨麻疹	Papular urticaria
	人工荨麻疹	Artificial urticaria
	急性荨麻疹	Acute urticaria
	过敏性荨麻疹	Allergic urticaria
	胆碱能性荨麻疹	Cholinergic urticaria
	慢性荨麻疹	Chronic urticaria
Symptom	荨麻疹图片	Urticaria pictures
	荨麻疹初期症状图片	Pictures of early symptoms of urticaria
	荨麻疹图片和症状	Urticaria pictures and symptoms
	荨麻疹症状	Urticaria symptoms
Etiology	荨麻疹会传染吗+荨麻疹传染吗	Is urticaria contagious
	荨麻疹是什么原因引起的+荨麻疹怎样引起的	What causes urticaria
	为什么会突然得了荨麻疹	Why am I getting urticaria all of a sudden
	为什么会得荨麻疹	Why do I get urticaria
Treatment	荨麻疹快速消退方法	How to get rid of urticaria symptoms fast
	荨麻疹怎么样治疗+荨麻疹治疗	How to treat urticaria
	荨麻疹怎么治疗最快的方法	The fastest way to cure urticaria
	荨麻疹怎么治疗能除根	How to eradicate urticaria
	荨麻疹五种食物不能吃	Five foods you can't eat when having urticaria
	荨麻疹吃什么药	What is the oral medicine of urticaria
	荨麻疹用什么药	What is the topical medicine for urticaria
	荨麻疹吃什么药最有效	What is the most effective oral medicine for urticaria

Supplementary Table 2. The average BSI and the average BSI difference in each province before and after COVID-19 pandemic.

Provinces	Before COVID-19 overall BSI	internet users × 10,000	Average BSI/10,000	After COVID-19 overall BSI	internet users × 10,000	Average BSI/10,000	Average BSI difference/10,000
Shandong	3,818,839	8855.1	431.26	5,309,024	8761.3	605.96	174.70
Guizhou	1,510,413	3520.1	429.08	2,219,486	3597.4	616.97	187.89
Jiangxi	1,939,251	3506.4	553.06	2,910,736	3599.3	808.70	255.63
Chongqing	1,862,328	3016.1	617.46	2,669,736	3117.2	856.45	238.99
Neimenggu	1,408,071	2606.2	540.28	2,089,716	2574.3	811.76	271.48
Hubei	2,497,461	4635.6	538.76	3,622,136	4789.7	756.23	217.48
Liaoning	2,518,065	4062.3	619.86	3,481,999	3921.8	887.86	268.00
Hunan	2,439,868	5489.7	444.44	3,640,029	5771.2	630.72	186.28
Fujian	2,624,329	3915.8	670.19	3,618,449	3979.6	909.25	239.06
Shanghai	2,569,731	3197.4	803.69	3,470,226	3365.7	1031.06	227.36
Beijing	3,017,249	3289.2	917.32	4,089,909	3272.9	1249.63	332.31
Guangxi	1,862,713	4450.2	418.57	2,844,527	4799.3	592.70	174.13
Guangdong	5,246,857	14200.3	369.49	7,315,154	14251.4	513.29	143.80
Sichuan	3,180,034	7277.7	436.96	4,460,478	7521.4	593.04	156.08
Yunnan	1,914,572	3829.3	499.98	2,742,798	3945.4	695.19	195.21
Jiangsu	4,088,648	8452.6	483.71	5,688,996	8428.7	674.96	191.24
Zhejiang	3,744,624	7047.0	531.38	5,138,818	7041.1	729.83	198.45
Qinghai	560,235	564.7	992.09	877,264	581.1	1509.66	517.57
Ningxia	680,207	685.7	991.99	1,047,689	712.7	1470.03	478.04
Hebei	2,863,226	6915.2	414.05	4,168,228	7104.0	586.74	172.70
Heilongjiang	1,808,611	2901.8	623.27	2,537,169	2980.3	851.31	228.04
Jilin	1,603,924	2304.8	695.91	2,286,202	2445.0	935.05	239.15
Tianjin	1,452,642	1450.7	1001.34	2,050,713	1488.8	1377.43	376.09
Shaanxi	2,149,756	3839.8	559.86	3,033,848	3876.2	782.69	222.82
Gansu	1,195,879	2323.1	514.78	1,759,448	2298.6	765.44	250.67
Xinjiang	1,221,115	2138.2	571.09	1,764,048	2359.2	747.73	176.64
Henan	3,221,841	8245.7	390.73	4,561,402	8748.7	521.38	130.65
Anhui	2,421,833	4790.8	505.52	3,715,172	5010.5	741.48	235.96
Shanxi	1,841,540	3142.3	586.05	2,766,222	3266.3	846.90	260.85
Hainan	954,628	931.5	1024.83	1,400,615	964.0	1452.92	428.09
Xizang	262,939	267.3	983.68	429,662	279.1	1539.46	555.77

Supplementary Table 3. The annual incidence rate of urticaria in 2020 and 2021 in the top six provinces with the largest increase in BSI before and after the COVID-19 pandemic.

**Provinces**	**annual incidence rate of COVID-19 in 2020 (per million population)**	**annual incidence rate of COVID-19 in 2021 (per million population)**
Xizang	0.27	0
Qinghai	3.04	2.03
Ningxia	10.41	6.53
Hainan	16.96	1.88
Tianjin	22.28	20.19
Beijing	45.08	10.23
